# Supplementary material for: Point singularity array with metasurfaces
Source: Nat Commun. 2023 Jun 5;14:3237. doi: 10.1038/s41467-023-39072-6 (PMC10241946; doi:10.1038/s41467-023-39072-6)
Supplement: Supplementary file 2 — Supplementary Information [file 41467_2023_39072_MOESM2_ESM.pdf]

Supplementary Information: Point singularity array with metasurfaces

Soon Wei Daniel Lim[1*†], Joon-Suh Park[1,2†], Dmitry Kazakov[1], Christina M. Spägele[1], Ahmed H. Dorrah[1], Maryna L. Meretska[1], Federico Capasso[1]

[1]Harvard John A. Paulson School of Engineering and Applied Sciences, 9 Oxford Street, Cambridge, MA 02138, USA
[2]Nanophotonics Research Center, Korea Institute of Science and Technology, Seoul 02792, Republic of Korea

*Email: lim982@g.harvard.edu
†Equal contribution

1. Computational design of metalens

The cylindrically symmetric phase-controlled metasurface at z=0 mm is parametrized by a set of 1001 annular rings, each of 500 nm radial extent, to produce a total lens with a 500 μm radius. For each radial position, we assign a scalar ϕ for the propagation phase delay of light there. This treats the metasurface as phase-only and cylindrically-symmetric. We then illuminate the phase-controlled surface with a uniform plane wave of vacuum wavelength 760 nm. This wavelength was chosen to be far blue-detuned from the D_2 resonance of Rubidium-87 atoms at 780.241 nm, which allows for the optical dipole potential to be written as directly proportional to the field intensity. This direct proportionality arises when the quantum-mechanical optical potential[1] is expanded to first order in the inverse detuning. We propagate this wavefront into the domain z>0 using the vectorial diffraction integral[2] implemented on an automatically differentiable platform (Tensorflow[3]). Automatic differentiation allows us to obtain objective function gradients efficiently and with a computational complexity that scales well with the number of degrees of freedom, which is 1001 in this problem. For concreteness, we assume that the incident polarization to the metasurface is oriented along the transverse x direction. The E_y and E_z components vanish on-axis by cylindrical symmetry (**Supplementary Figure 7**). For intensity calculations off-axis, we use all three Cartesian components.

The 0D singularity positions are located at $r = 0$ and $z_i = 500\ \mu\text{m}, 503\ \mu\text{m}, \ldots, 527\ \mu\text{m}$ and are uniformly spaced 3 μm apart. The uniform spacing is not necessary; the positions can have any spacing along the longitudinal axis (**Supplementary Figure 1**). However, the light intensity between adjacent singular positions will become small if the spacing is comparable to or smaller than the characteristic longitudinal extent of focused spots, which can be approximated by the depth of focus λ/NA^2, where NA is the equivalent numerical aperture for a focusing lens with a focal spot at that longitudinal position. In this system, the equivalent depth of focus given the system NA is approximately 1.5 μm, which is smaller than the desired singularity spacing. The singularity spacings in this case are chosen to be close to that of prior ^{87}Rb Rydberg atom arrays[4].

We design the 0D singularity array using two stages of numerical optimization. These optimizations are performed with respect to the 1001 phase values over the cylindrically-symmetric metasurface. In the first stage of optimization, we maximize the longitudinal phase

gradient at the target singularity positions. This step produces a 0D singularity at each of the target singularity positions. In the second stage of optimization, we use the optimized first stage result to equalize the phase gradient and second spatial derivative of the field intensity over all the singularity positions and thus obtain nearly identical singularities across the array.

For the first optimization stage, at each singularity position, we compute the z-directed phase gradient of the field $\partial\phi/\partial z$. The objective function F_1 to be minimized is the negative minimum of the squares of the z-directed phase derivatives for each singularity position:

$$F_1 = -\min\{(\partial\phi/\partial z_i)^2\}_{i=1,\ldots,n}$$

To improve convergence, we use a smooth approximation to the minimum function, which is analytic instead of being piecewise continuous:

$$\min(a_1, \ldots, a_n) = \frac{\log[\sum_i \exp(-s \cdot a_i)]}{-s}, s = \frac{100}{\frac{1}{n}\sum_i |a_i|} > 0$$

The sum inside the argument of the logarithm is dominated by the term corresponding to smallest value of a_i. s is a scale factor chosen to bring the input array values onto the same approximate scale and avoid numerical loss of precision during the computation of the exponential.

In the second optimization stage, the objective function F_2 to be minimized is the maximum of the deviations of the phase gradient to a large target phase gradient, set here to be 100 times the nominal field wavenumber k_0, plus penalty terms for differences in the second spatial derivative of the on-axis intensity $I(z) = |E_x(r = 0, z)|^2$:

$$F_2 = \max\left(\frac{\partial\phi}{\partial z_i} - 100k_0\right)^2_{i=1,\ldots,n} + c_1 \frac{\sigma\{\partial_z^2 I(z_i)\}_{i=1,\ldots,n}}{\mu\{\partial_z^2 I(z_i)\}_{i=1,\ldots,n}} + c_2 \frac{\sigma\{\partial_r^2 I(z_i)\}_{i=1,\ldots,n}}{\mu\{\partial_r^2 I(z_i)\}_{i=1,\ldots,n}}$$

where σ refers to the population standard deviation and μ is the population mean. c_1 and c_2 are weight parameters that are chosen so as to bring the three terms in F_2 onto similar scales. We use a smooth approximation to the maximum function to improve convergence, which is analogous to the smooth approximation to the minimum function described earlier.

$$\max(a_1, \ldots, a_n) = \frac{\log[\sum_i \exp(s \cdot a_i)]}{s}, s = \frac{100}{\frac{1}{n}\sum_i |a_i|} > 0$$

We find that F_2 convergence can be improved by ramping up the target phase gradient from around $5k_0$ to $100k_0$ in the objective function. That is, using the converged results from step 1, we target a phase gradient of $5k_0$ in F_2, find a local optimization minimum, then repeat the process for a higher target phase gradient until we reach the target of $100k_0$.

In order to realize this optimized radial phase profile in a metasurface that operates in transmission, we seek to place a meta-atom at each radial position (spaced in the circumferential direction by the meta-atom pitch of 500 nm) to enforce the required phase at that radial position. This nanostructure is chosen from a library of meta-elements comprising nanopillars made of 700 nm-

tall amorphous TiO_2 mounted on a substrate of fused silica. These meta-atoms are shown schematically in **Supplementary Figure 11(a)** and the dependence of the phase and transmission efficiency as a function of the nanopillar diameter is plotted in **Supplementary Figure 11(b)**. Meta-elements close to resonances are removed from the library. Nanopillars of diameter between 80 nm and 480 nm provide 2π phase coverage and are thus used to construct the metasurface based on the required phase profile. The library was computed using rigorous coupled wave analysis on the RETICOLO[5] v8 platform.

2. Comparison with Intensity Gradient optimization

To make a comparison of *z*-directed phase gradient maximization with *z*-directed intensity gradient maximization, we performed an identical optimization to that of section 1 with the only difference being the use of the on-axis intensity gradient for the objective function:

$$F_1 = -\min\{(\partial|\mathrm{E_x}|^2/\,\partial \mathrm{z_i})^2\}_{\,i=1,\ldots,\mathrm{n}}$$

Since the other polarization components $E_{y,z}$ vanish on axis, the total intensity is only a function of the *x*-polarized electric field for *x*=*y*=0. **Supplementary Fig. 10a-b** exhibits the *xz* intensity profile after Step 1 optimization, in which the *z*-directed intensity gradients at ten equally-spaced on-axis positions from z=500 um to z=527 um are maximized. The tunable parameters are the same as in the device of section 1: the 1001 phase values on the metasurface radial profile. The resultant intensity and phase gradients along the optical axis (x=y=0) are also plotted in **Supplementary Fig. 10c**.

We notice that the dark regions are non-systematically displaced in the z-direction from the positions of intensity gradient maximization, and that the phase gradients in the adjacent dark regions are not appreciably larger in magnitude than the vacuum wavenumber k_0. However, the absolute values of the intensity are substantially larger than those produced by phase gradient maximization due to most field structure being concentrated near the optimization points, whereas the device obtained through phase gradient maximization produces field structure further away from the optimization points, thereby spreading out the wave energy. Intensity maximization may be useful for applications which are less sensitive to the precise positioning of the dim regions or which emphasize contrast between the dark and bright regions.

3. Experimental characterization of point singularity array

Figure 3d shows optical and scanning electron micrographs (Zeiss UltraSEM) of a singularity array metasurface processed under identical conditions to the metasurface used for optical characterization, respectively. The metasurface used for optical characterization was not imaged in the SEM because this requires the irreversible deposition of a conductive metallic layer. The experimental setup for characterizing the singularity array metasurface is depicted in **Figure 3e**. A 760.9 nm single frequency distributed feedback (DFB) laser (TOPTICA Eagleyard GmbH) is driven with a constant current source (Newport 505 Laser Diode Driver) and kept at a constant temperature (Newport 325 Thermoelectric Cooler Driver). The single mode fiber-coupled output is collimated with a reflective collimator (Thorlabs RC12APC-P01) and is incident on the fused silica face of the metasurface. The metasurface *z*-position is controlled using a closed-loop piezo-

motor stage with nm resolution (Attocube ECSx3030). The transmitted light is captured using a horizontal microscope system comprising a high NA objective (Olympus 100x MPLAPON NA=0.95), tube lens (Thorlabs TTL-180A) and CMOS camera (Thorlabs DCC1545M). The intensity image is captured over a range of longitudinal z-positions at steps of 50 nm, where z=0 mm corresponds to the patterned surface of the metasurface. At each z-position, the system is allowed to stabilize for 10 seconds before multiple intensity images are captured at different exposure times ranging from 0.05 ms to 163 ms. These multiple exposure images are later weighted by their respective exposure times and stacked to remove saturated pixels and produce a composite image with a large intensity dynamic range.

To set an absolute power scale for the transmitted light field, we measure the incident and transmitted energy flux. The incident power is measured through a 1 mm pinhole (Thorlabs P1000K) using a silicon power sensor (Thorlabs S120B). The 1 mm diameter is chosen to match the diameter of the metasurface. The transmitted energy flux is measured indirectly by estimating the power flowing through each pixel of the CMOS sensor at the axial plane of maximum on-axis intensity, which occurs at around z = 530 μm. This is measured by placing an iris and power meter head between the tube lens and the CMOS sensor. The iris is used to reduce the diameter of the light beam incident on the CMOS sensor so that it fits entirely within the sensor area. By making the approximation that the intensity recorded by each pixel on the CMOS sensor (with the power meter removed) is proportional to the energy flux through that pixel, we are thus able to relate the intensity distribution recorded on the CMOS sensor to the total power flux recorded by the power meter head. The CMOS sensor image is captured at a high intensity dynamic range using a range of exposure times from 0.08 ms to 245 ms to improve the estimation precision. The power flux through the maximum intensity pixel on the transverse plane is used to set the absolute power scale for the z-stack measurements in **Figure 4**. Note that the experimental intensity measurements are an underestimation of the true intensity values since the transmitted intensities are measured after the microscope objective and the tube lens, which introduce reflective losses.

4. Numerical characterization of the point singularity array as a blue trap array

The light distribution in the vicinity of each singular point in the experimental array is characterized by fitting the volumetric light distribution to second-order polynomials. The axial location of each singular point is first determined by fitting the on-axis intensity in a 1D window (1 μm full width) around each singular point and estimating the minimum intensity axial position using the fitted coefficients. The fitted quadratic coefficient provides an estimate of the curvature of the on-axis intensity profile. The transverse intensity profile at each singular point axial profile is then fitted to a 2D quadratic polynomial using a window width of 1 μm in both transverse directions. The fitted quadratic coefficients yield the intensity profile curvature in the transverse directions.

The measured intensity profile $I(\boldsymbol{r})$ can be converted to optical potential values $U(\boldsymbol{r})$ in the context of neutral atom dipole traps by the relationship[1]:

$$U(\boldsymbol{r}) = \frac{\hbar\delta}{2}\log\left[1 + \frac{\frac{I(\boldsymbol{r})}{I_{sat}}}{1 + \left(\frac{2\delta}{\Gamma}\right)^2}\right]$$

$\delta = \omega - \omega_0$ is the detuning of the trap frequency to the dipole resonance frequency, I_{sat} is the saturation intensity, and Γ is the natural linewidth of the dipole transition. For the D_2 line of ^{87}Rb, $\omega_0 = c\left(\frac{2\pi}{780.241\text{ nm}}\right) = 2\pi \cdot 384.230\text{ THz}, \Gamma = 2\pi \cdot 6.0666\text{ MHz}, I_{sat} = 2.50399\text{ mW/cm}^2$ [6]. In the limit where $I(\boldsymbol{r}) \ll I_{sat}\left[1 + \left(\frac{2\delta}{\Gamma}\right)^2\right]$ and $\left(\frac{2\delta}{\Gamma}\right)^2 \gg 1$, the argument of the logarithm can be Taylor expanded to first order so that the optical potential is linear in $I(\boldsymbol{r})$:

$$U(\boldsymbol{r}) = \frac{\hbar\delta}{2}\frac{\frac{I(\boldsymbol{r})}{I_{sat}}}{\left(\frac{2\delta}{\Gamma}\right)^2}$$

The curvature of the optical potential around the singularity is directly connected to the effective spring constant of an atom placed at the center of the singular position:

$$k_i = \frac{\partial^2 I(\boldsymbol{r})}{\partial i^2}, i = x, y, z$$

The trap frequency in each direction can then be calculated from the spring constants and the atom mass m.

$$\omega_i = \frac{1}{2\pi}\sqrt{\frac{k_i}{m}}$$

The trap depth for each point singularity is computed by taking the optical potential difference between the optical potential at the center of the trap and that of the first potential peak encountered when moving away from the center of the trap. Since the trap is not spherically symmetric, this potential difference will depend on the direction at which one moves away from the trap center. We introduce a polar coordinate system with the polar axis aligned with the *z*-direction of the optical axis. The azimuthal angle is the direction corresponding to cylindrical symmetry. For each polar and azimuthal angle, we draw a line emanating from the trap center and pointing in that direction. We then compute the optical potential along that line and identify the potential barrier as the height of the first peak encountered relative to the potential at the trap center. We quantify the trap depth as a function of the polar angle θ with respect to the optic axis by computing the trap depth for the 2π azimuthal angle range with the same polar angle, then taking the minimum (worst case) trap depth. These potential depths are plotted as a function of θ in **Supplementary Figure 15c-d**. The trap depth is nonvanishing for all polar angles, indicating that every trap obtained numerically and experimentally has 3D confinement. The overall trap depth for each trap can then be associated with the minimum trap depth over all polar angles. This overall trap depth is at least 1.87 mK/W for the simulated optical potential profile and is measured to be at least 0.22

mK/W for the experimental optical potential profile. The units of the trap depth are chosen to be in temperature units (through division with the Boltzmann constant k_B) and are normalized to the incident power on the metalens.

5. Incident tilt and chromatic dependence of singularity array

In **Supplementary Figure 16**, we examine the chromatic and incident beam tilt dependence of the singularity array metasurface on trapping behavior, partitioning the performance based on the trap index within the array from 1 to 10. These studies are performed numerically using the metasurface geometry and material optical parameters and assume a fixed 1W incident trapping power over the 1 mm metasurface. Under large chromatic shift or incident tilt, several trap positions lose 3D confinement and are not plotted in **Supplementary Figure 16**. Changing the incident trap wavelength changes the realized phase and amplitude profile of the metasurface in a manner similar to that of diffractive optics: the effective focal length of the metasurface decreases as the wavelength increases, causing the structured intensity pattern to translate along the optical axis with minor perturbation. The minimum potential depth is largely dependent on the detuning from the D_2 line and increases with decreasing detuning (**Supplementary Figure 16a**), although the variation in trap depths increases at smaller detuning as well. The trap center scattering rate, which depends on the field intensity at the trap center, remains low with small variations over trap positions for further detuning, but becomes larger and with high variation for reduced detuning (**Supplementary Figure 16c**). The axial and radial trapping frequency chromatic dependencies which are obtained by quadratic fitting of the trap potential in a 1 μm diameter window around each trap to obtain the potential curvature, are plotted in **Supplementary Figure 16e** and **g**, respectively. The inter-trap variation in trapping frequency is minimized at the design wavelength. The movie of the field structure and trap positions as a function of increasing wavelength is included as **Supplementary Movie 1**.

The tilt dependence of the trap array is of interest because of the potential use of metasurface traps in tweezer arrays. Tweezer arrays are currently able to produce multiple red-detuned trap positions by diffracting an incident trap laser into multiple outgoing tilt angles using acousto-optic deflectors[4,7,8]. Each diffraction order has a beam tilt that controls the transverse displacement of the focal spot from the optical axis when imaged into the vacuum cell using a high NA objective. One may consider "duplicating" the blue trap array by illuminating the metasurface with a number of trap lasers at different tilt angles, thereby producing one copy of the array at different transverse displacements. Our metasurface is not designed for off-axis illumination and thus shows a rapid fall-off in potential depth as a function of incident beam tilt angle (**Supplementary Figure 16b**), falling to half the potential depth at an angle of 2 mrad (0.11°). The trap center scattering rate increases nonlinearly with the beam tilt (**Supplementary Figure 16d**). The axial trapping frequency remains relatively stable with tilt (**Supplementary Figure 16f**), but the radial trapping frequency falls off more rapidly and is the reason behind several traps losing 3D confinement (**Supplementary Figure 16h**). The movie of the field structure and trap positions as a function of increasing beam tilt is included as **Supplementary Movie 2**.

Supplementary Figures

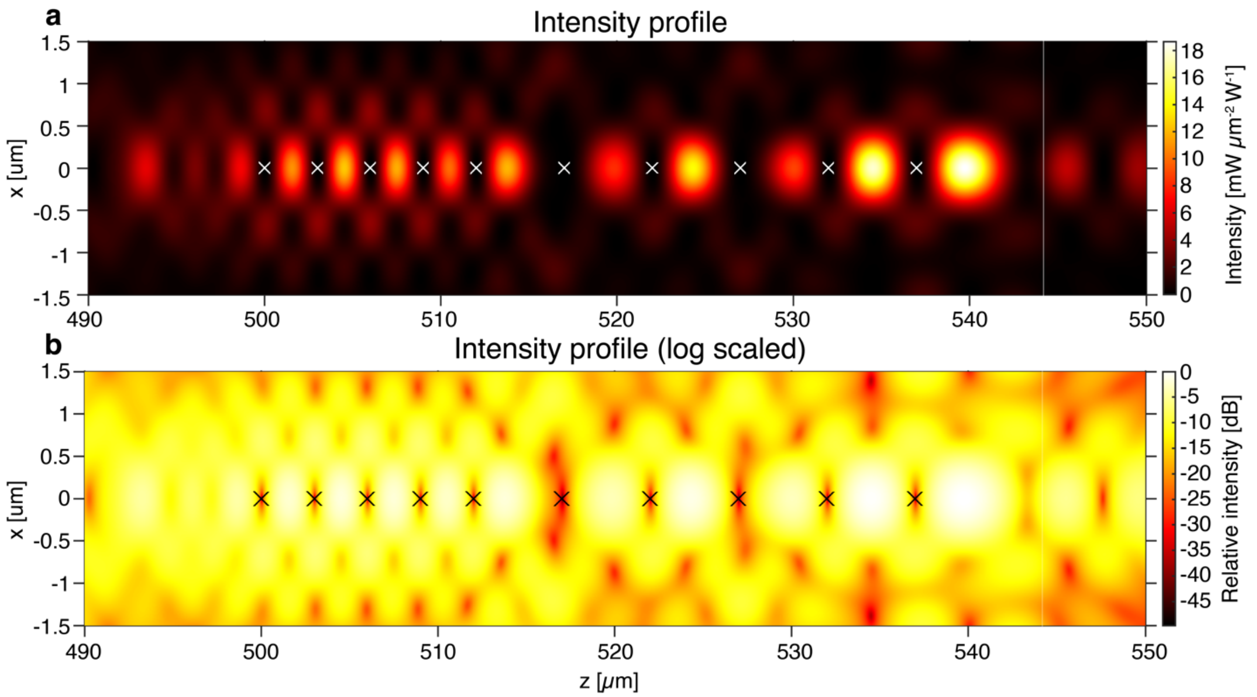

Supplementary Figure 1. Demonstration of a non-uniformly-spaced 0D singularity array. (**a**) *xz* intensity plot of a cylindrically symmetric 0D singularity array with five singularities spaced 3 µm apart (z = 500 µm to z = 512 µm) and five singularities spaced 5 µm apart (z = 517 µm to z = 537 µm). Crosses indicate the positions of the 0D singularities at which the phase gradient optimization was performed. (**b**) Logarithmically-scaled intensity plot of **a**.

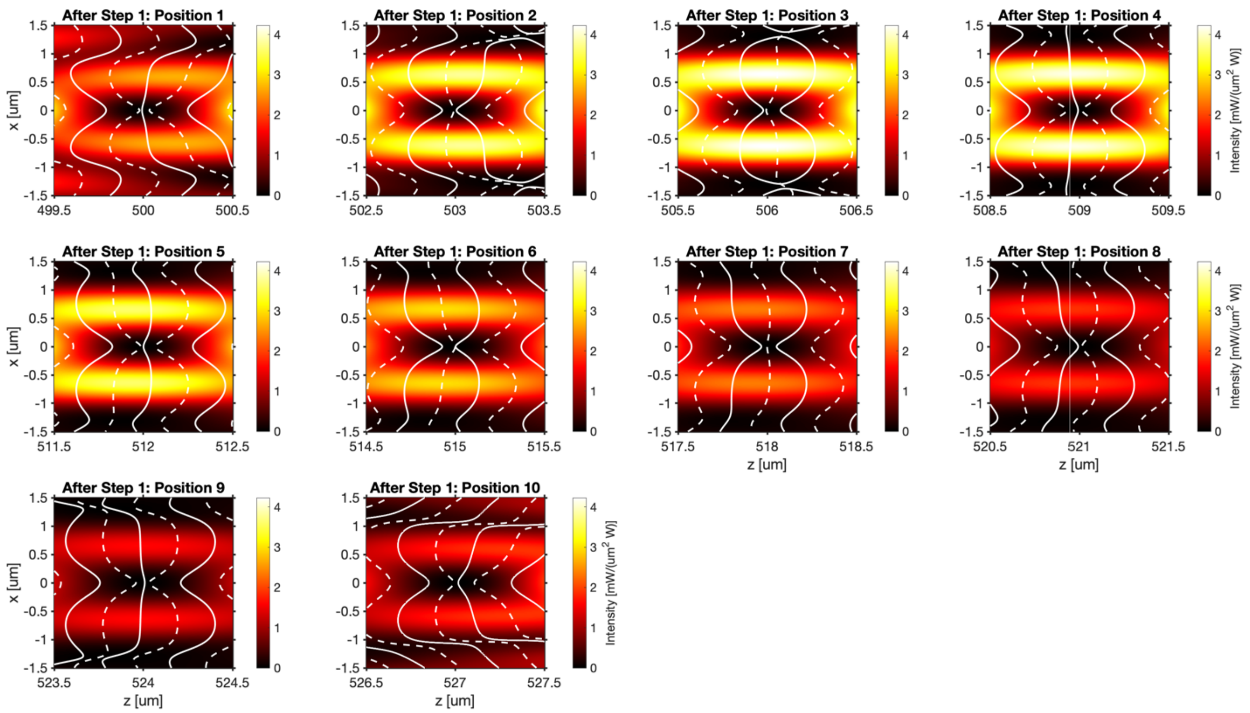

Supplementary Figure 2. Intensity $|E_x|^2+|E_y|^2+|E_z|^2$ profile and zero-isolines for the ten 0D singularity positions in the array, just after the first optimization step. Zero-isolines for the real part of the scalar field where Re(E_x)=0 are plotted as solid black lines; zero-isolines for the imaginary part of the scalar field where Im(E_x)=0 are plotted as dashed black lines. The amplitude profile at the metasurface plane is assumed to be uniform in this calculation.

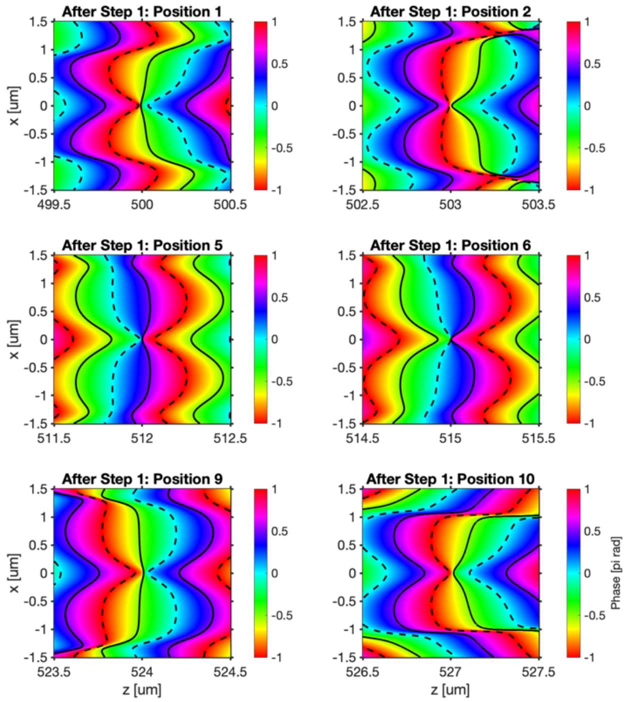

Supplementary Figure 3. E_x phase profile and zero-isolines for the ten 0D singularity positions in the array, just after the first optimization step. Zero-isolines for the real part of the scalar field where Re(E_x)=0 are plotted as solid black lines; zero-isolines for the imaginary part of the scalar field where Im(E_x)=0 are plotted as dashed black lines. The amplitude profile at the metasurface plane is assumed to be uniform in this calculation.

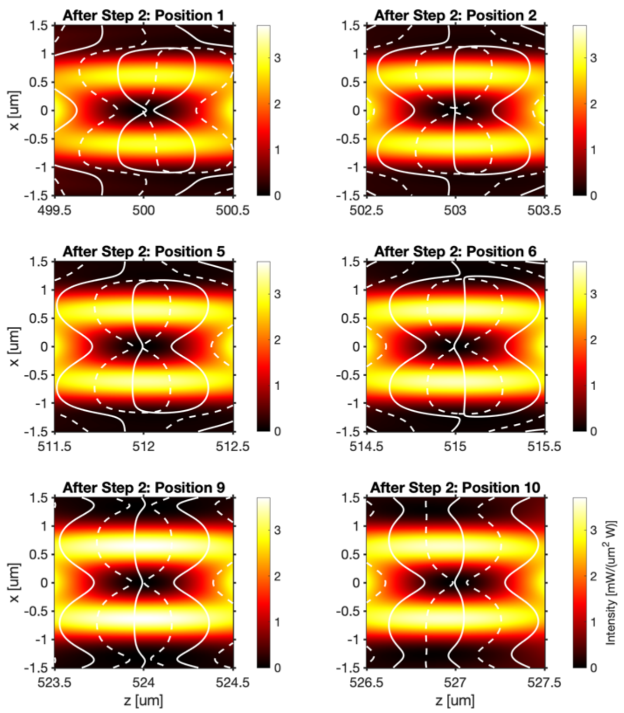

Supplementary Figure 4. Intensity $|E_x|^2+|E_y|^2+|E_z|^2$ profile and zero-isolines for the ten 0D singularity positions in the array, just after the second optimization step. Zero-isolines for the real part of the scalar field where Re(E_x)=0 are plotted as solid black lines; zero-isolines for the imaginary part of the scalar field where Im(E_x)=0 are plotted as dashed black lines. The amplitude profile at the metasurface plane is assumed to be uniform in this calculation.

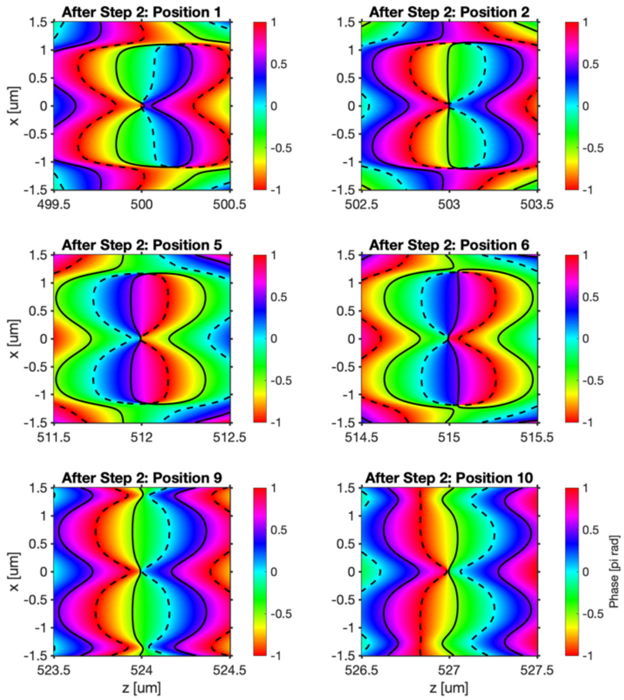

Supplementary Figure 5. E_x phase profile and zero-isolines for the ten 0D singularity positions in the array, just after the second optimization step. Zero-isolines for the real part of the scalar field where Re(E_x)=0 are plotted as solid black lines; zero-isolines for the imaginary part of the scalar field where Im(E_x)=0 are plotted as dashed black lines. The amplitude profile at the metasurface plane is assumed to be uniform in this calculation.

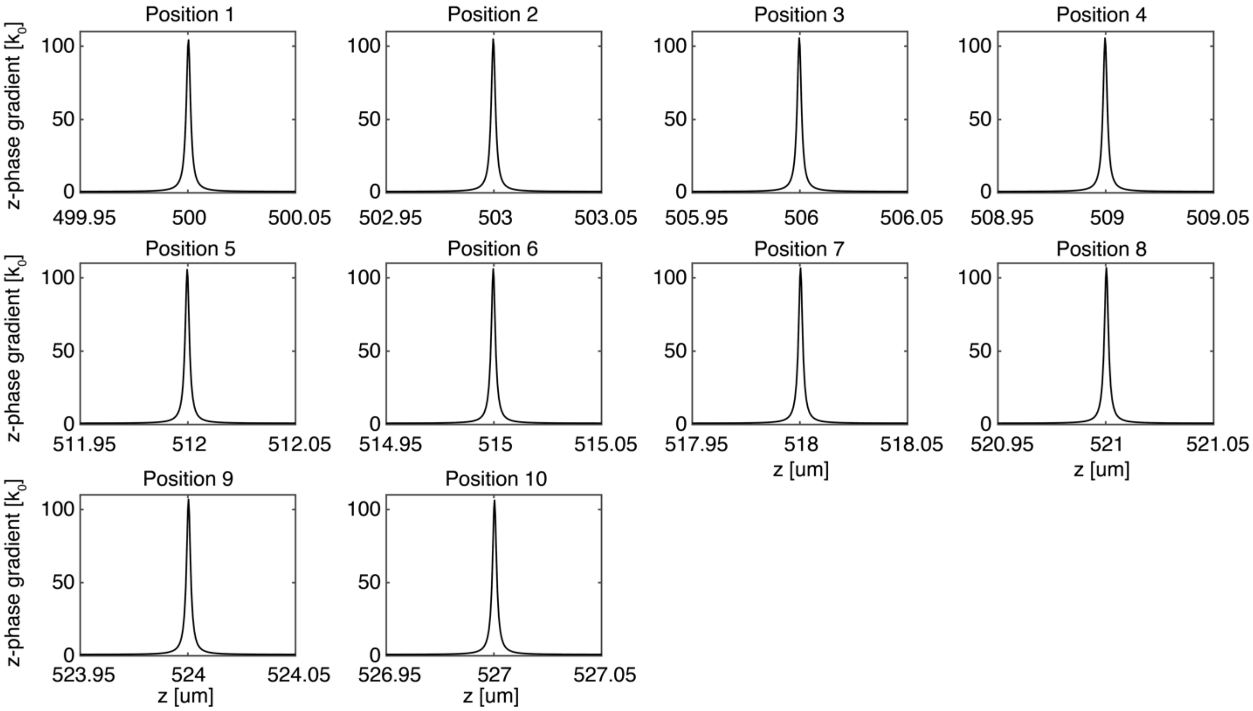

Supplementary Figure 6. Numerically-calculated *z*-directed phase gradient in the vicinity of each of the ten singularity locations. The full-width-at-half-maximum of the phase gradient is 2.3 nm for every location.

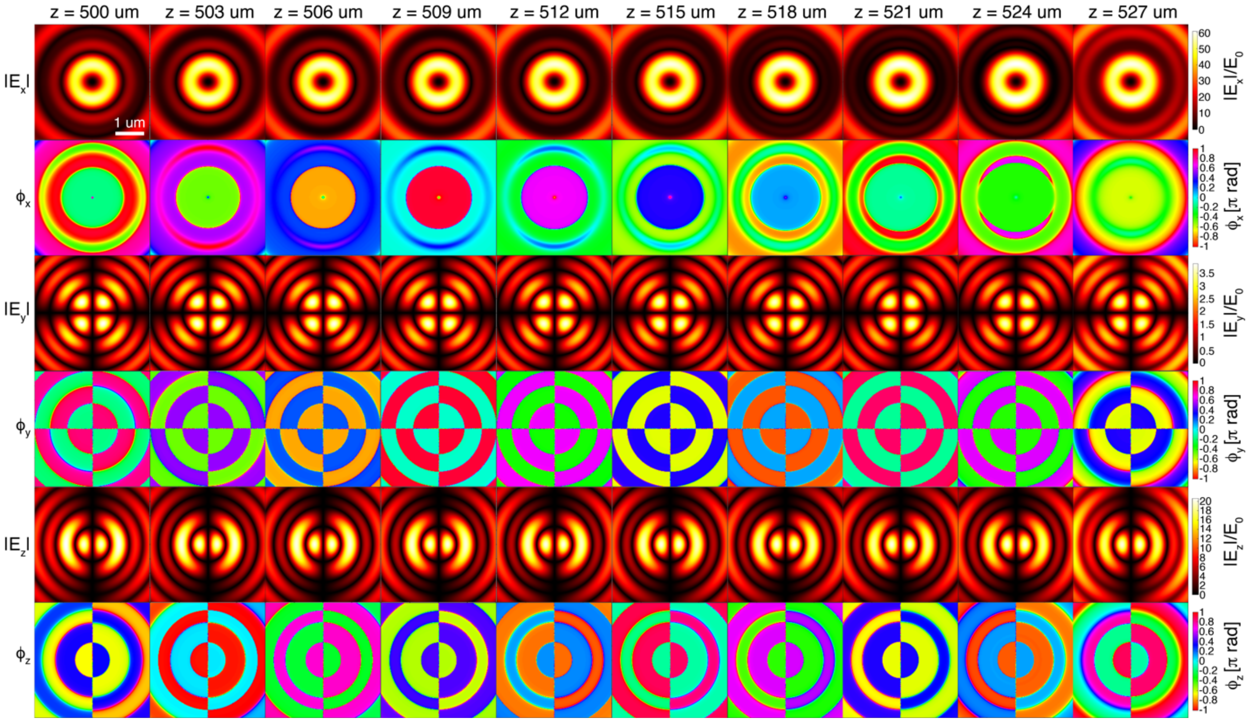

Supplementary Figure 7. Cross-sectional *xy* cuts for the Cartesian field components (E_x, E_y, E_z) after the second optimization step. The electric field values are normalized to E_0, the incident *x*-polarized electric field magnitude at the metasurface. Rows from top to bottom: Magnitude of E_x, phase of E_x, magnitude of E_y, phase of E_y, magnitude of E_z, phase of E_z. The plots are centered on (x=0, y=0).

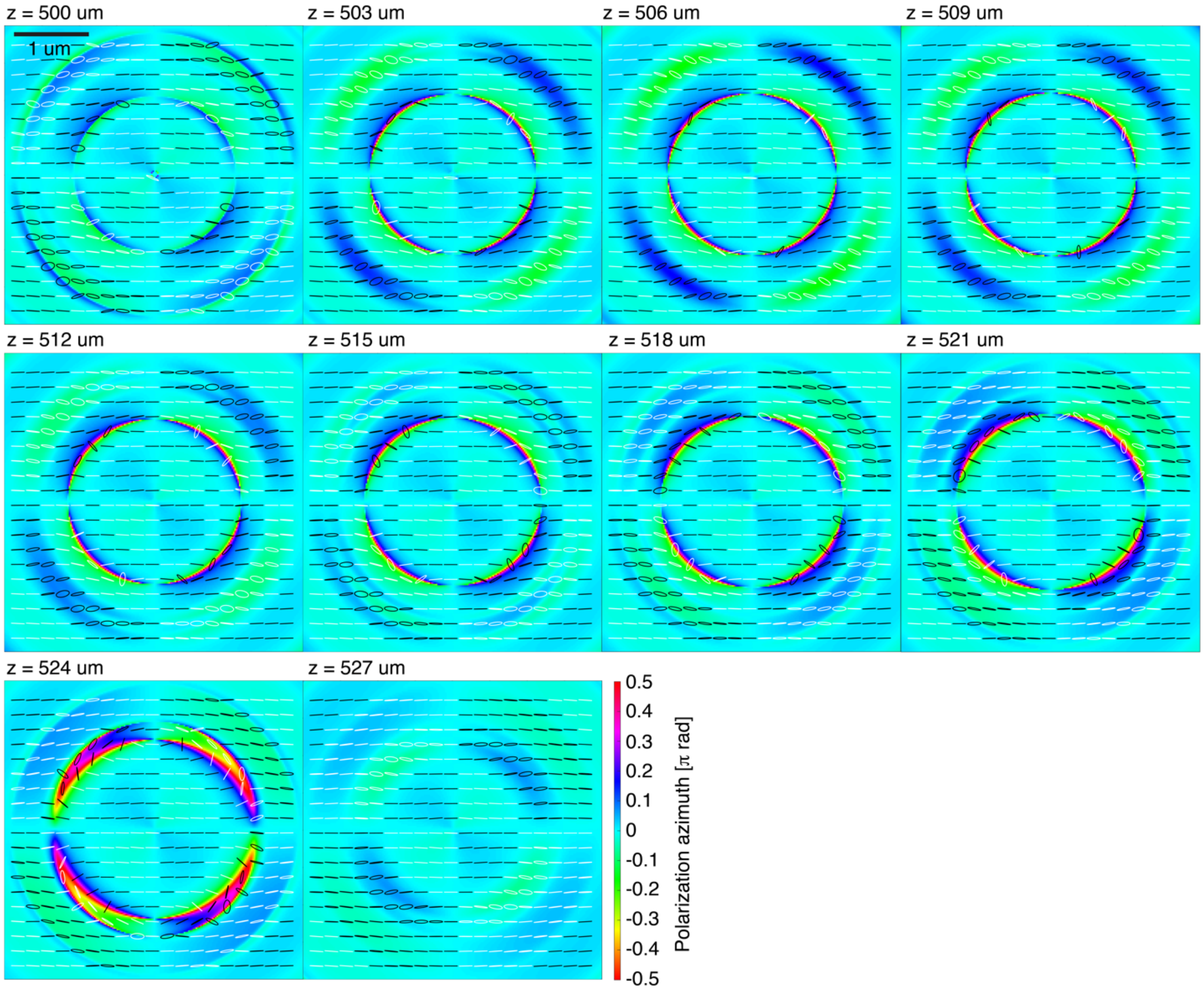

Supplementary Figure 8. Cross-sectional *xy* cuts of the polarization azimuth (surface plot) and polarization ellipses (superimposed) after the second optimization step. The metasurface is illuminated with *x*-polarized light. Black ellipses indicate right elliptical/circular polarization and white ellipses indicate left elliptical/circular polarization. The plots are centered on (*x*=0, *y*=0).

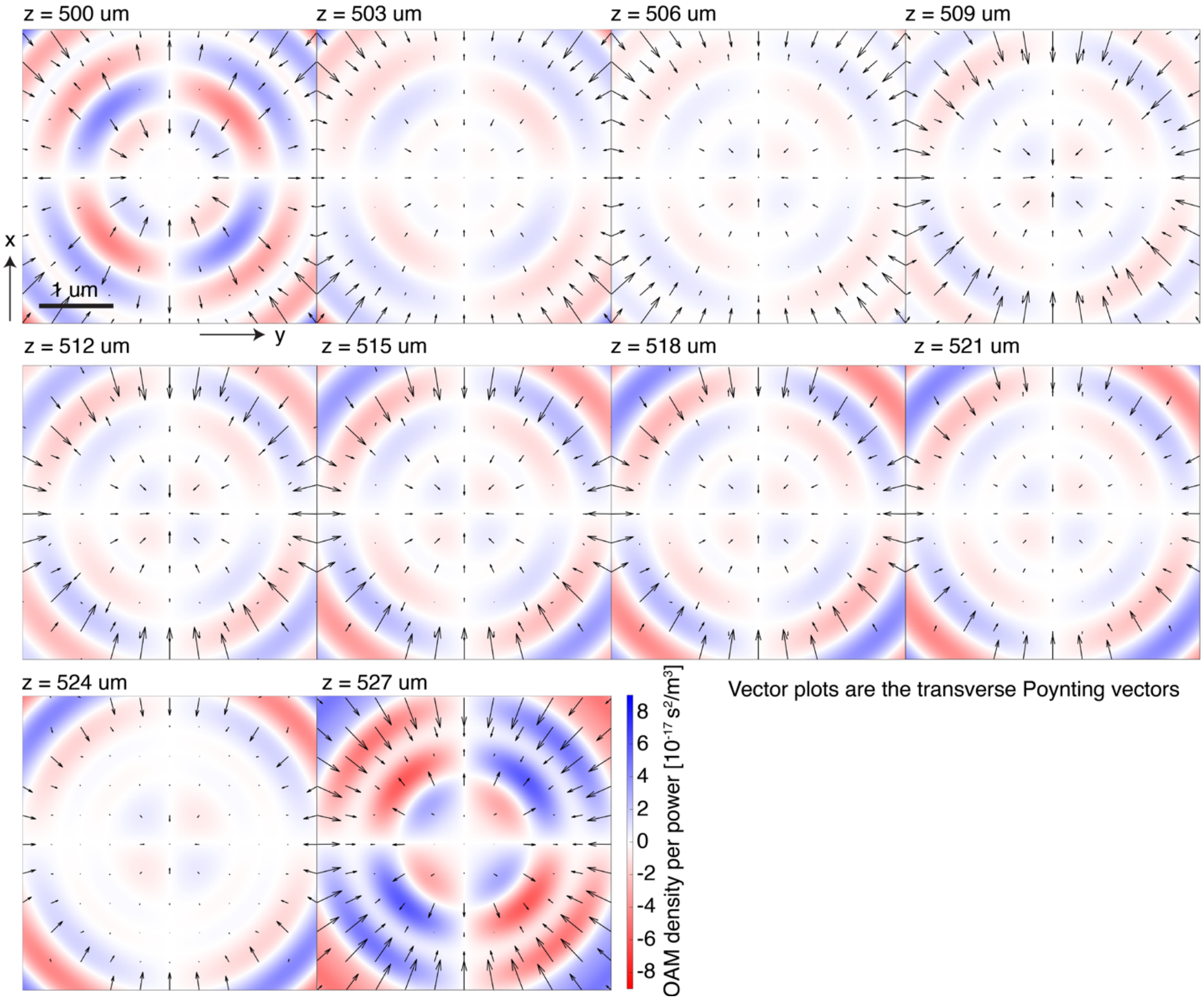

Supplementary Figure 9. Transverse *xy* plot of numerically-simulated orbital angular momentum (OAM) density and power flux, after the second optimization step, at each singularity plane. The metasurface is illuminated with *x*-polarized light. The surface plot is the orbital angular momentum per incident power at the metasurface and the vector plot is the transverse projection of the Poynting vector. The plots are centered on the optic axis (*x*=0, *y*=0). The tiny OAM densities and lack of azimuthal circulation about the optic axis demonstrate that the OAM contributions are negligible in this system.

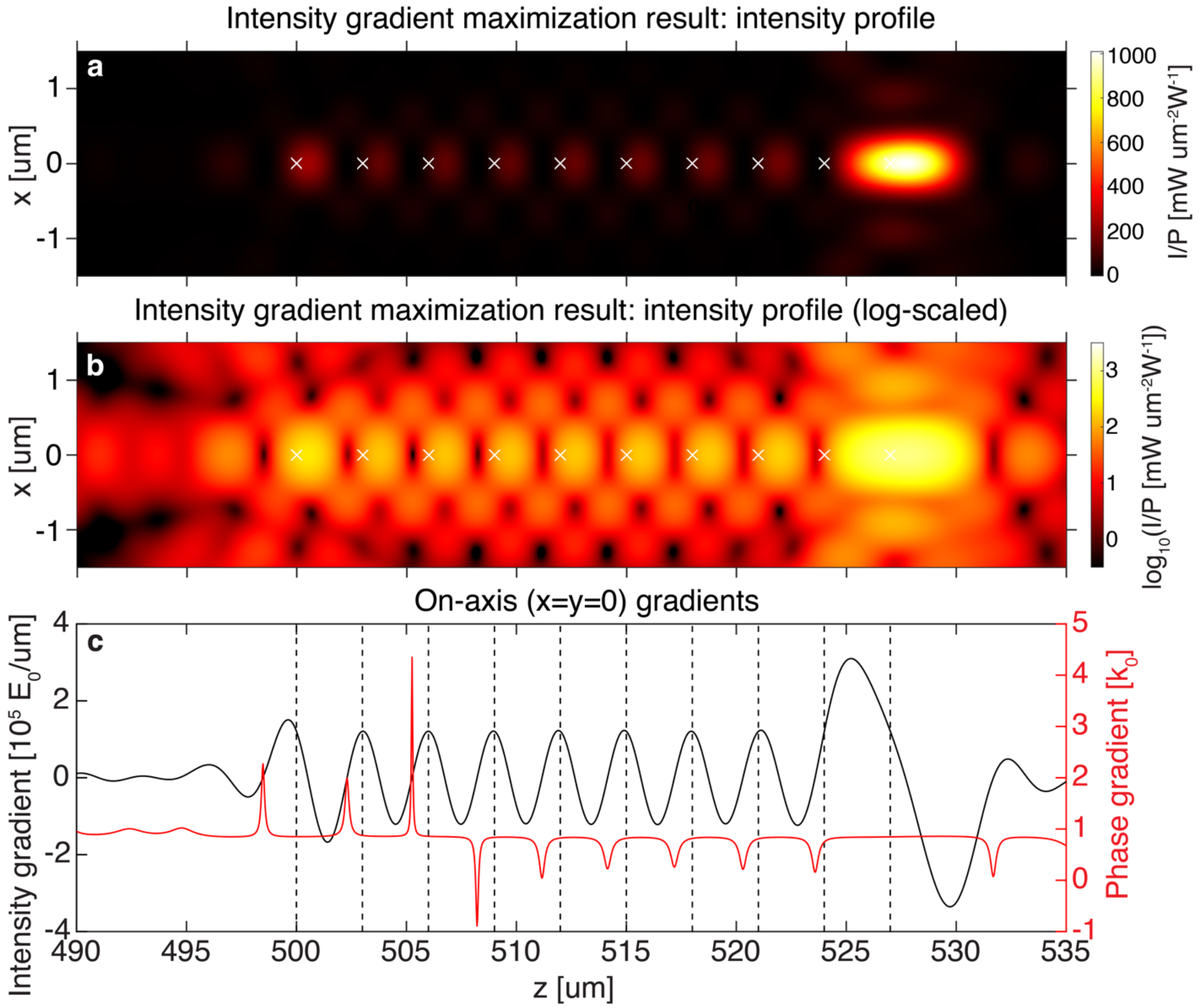

Supplementary Figure 10. Array designed using *z*-directed intensity gradient maximization. **(a)** *xz* total intensity profile, white crosses are the positions at which the intensity gradient was maximized. (**b)** log-scaled *xz* total intensity profile. **(c)** Intensity and phase gradient profile along the optical axis (x=y=0). E_0 is the incident electric field at the metasurface and k_0 is the vacuum wavenumber. The vertical dotted lines are the positions at which the intensity gradient was maximized. The maximum phase gradient position and the minimum intensity positions are displaced in an inconsistent fashion from the peak intensity gradient positions.

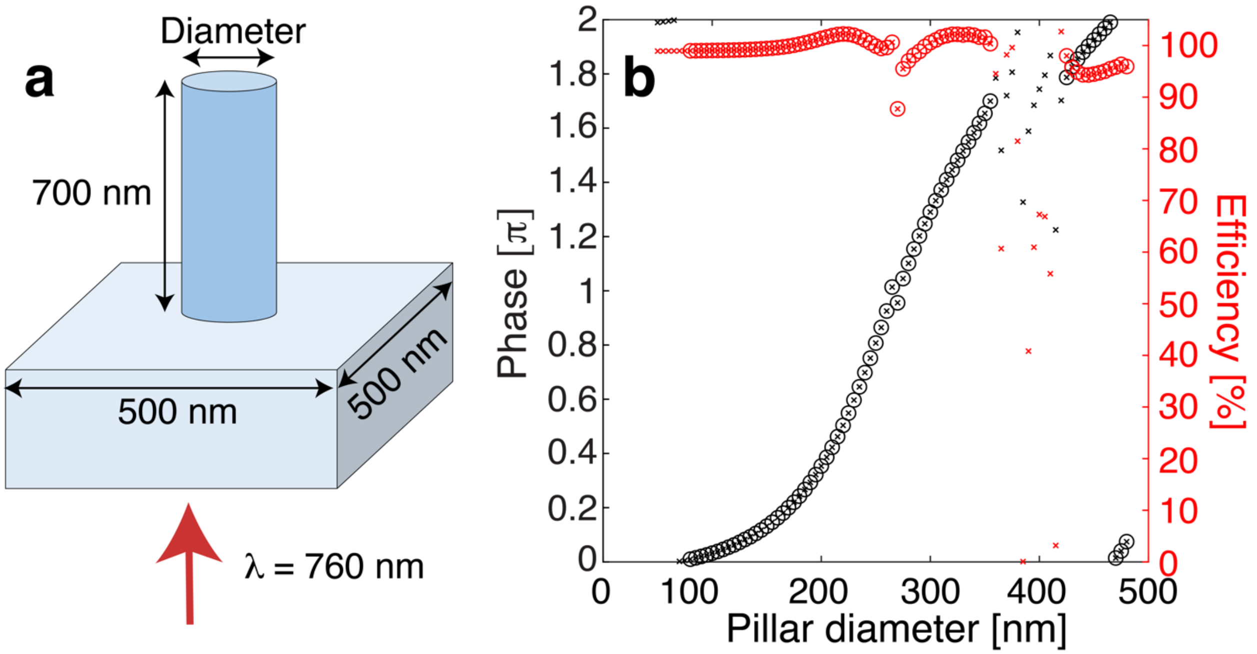

Supplementary Figure 11. (**a**) Cylindrical meta-atom geometry used in realizing the 0D singularity array. (**b**) Transmission phase and efficiency dependence on the nanopillar diameter for the cylindrical meta-atom. The circled data points are used in the meta-atom library. The diameter range used is 80 nm to 480 nm, which provides 2π phase coverage.

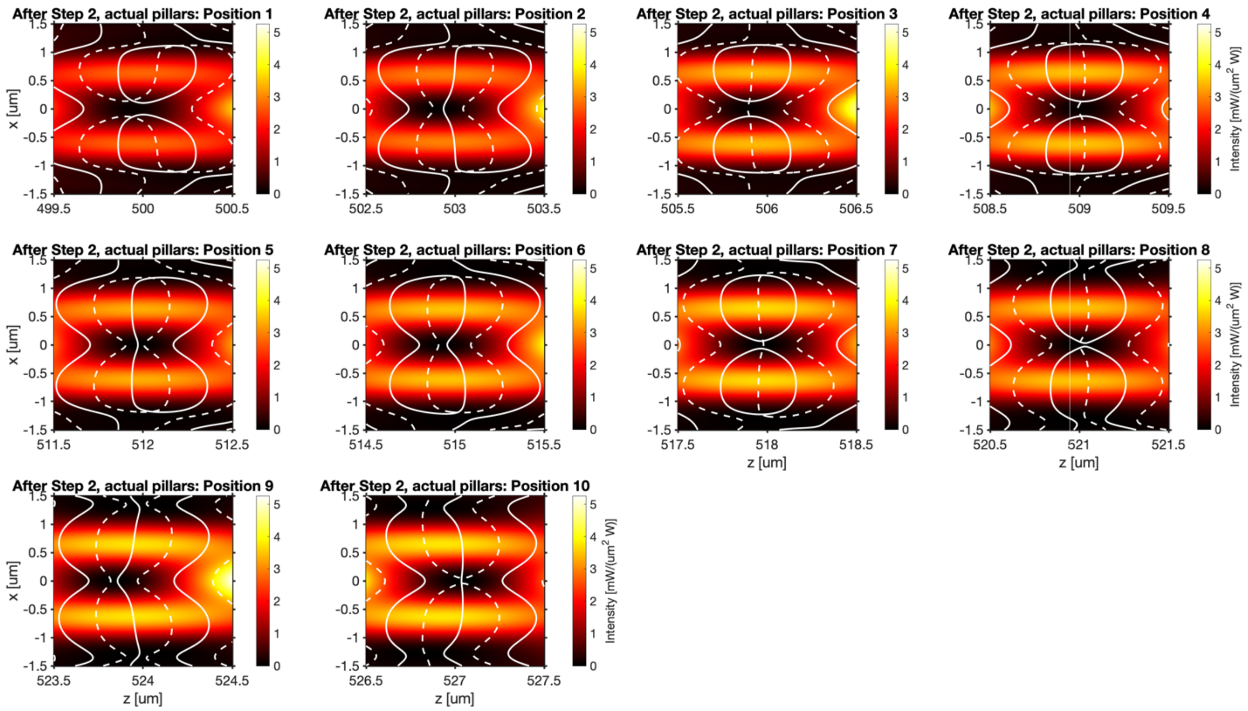

Supplementary Figure 12. Intensity $|E_x|^2+|E_y|^2+|E_z|^2$ profile and zero-isolines for the ten 0D singularity positions in the array, after the second optimization step, and incorporating the non-uniform transmission amplitudes of the TiO_2 nanopillar library. Zero-isolines for the real part of the scalar field where Re(E_x)=0 are plotted as solid black lines; zero-isolines for the imaginary part of the scalar field where Im(E_x)=0 are plotted as dashed black lines.

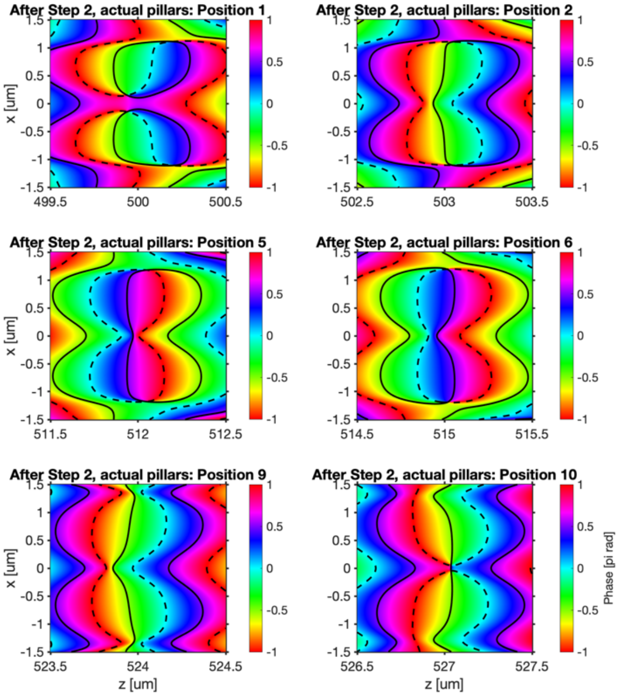

Supplementary Figure 13. E_x phase profile and zero-isolines for the ten 0D singularity positions in the array, after the second optimization step, and incorporating the non-uniform transmission amplitudes of the TiO_2 nanopillar library. Zero-isolines for the real part of the scalar field where Re(E_x)=0 are plotted as solid black lines; zero-isolines for the imaginary part of the scalar field where Im(E_x)=0 are plotted as dashed black lines.

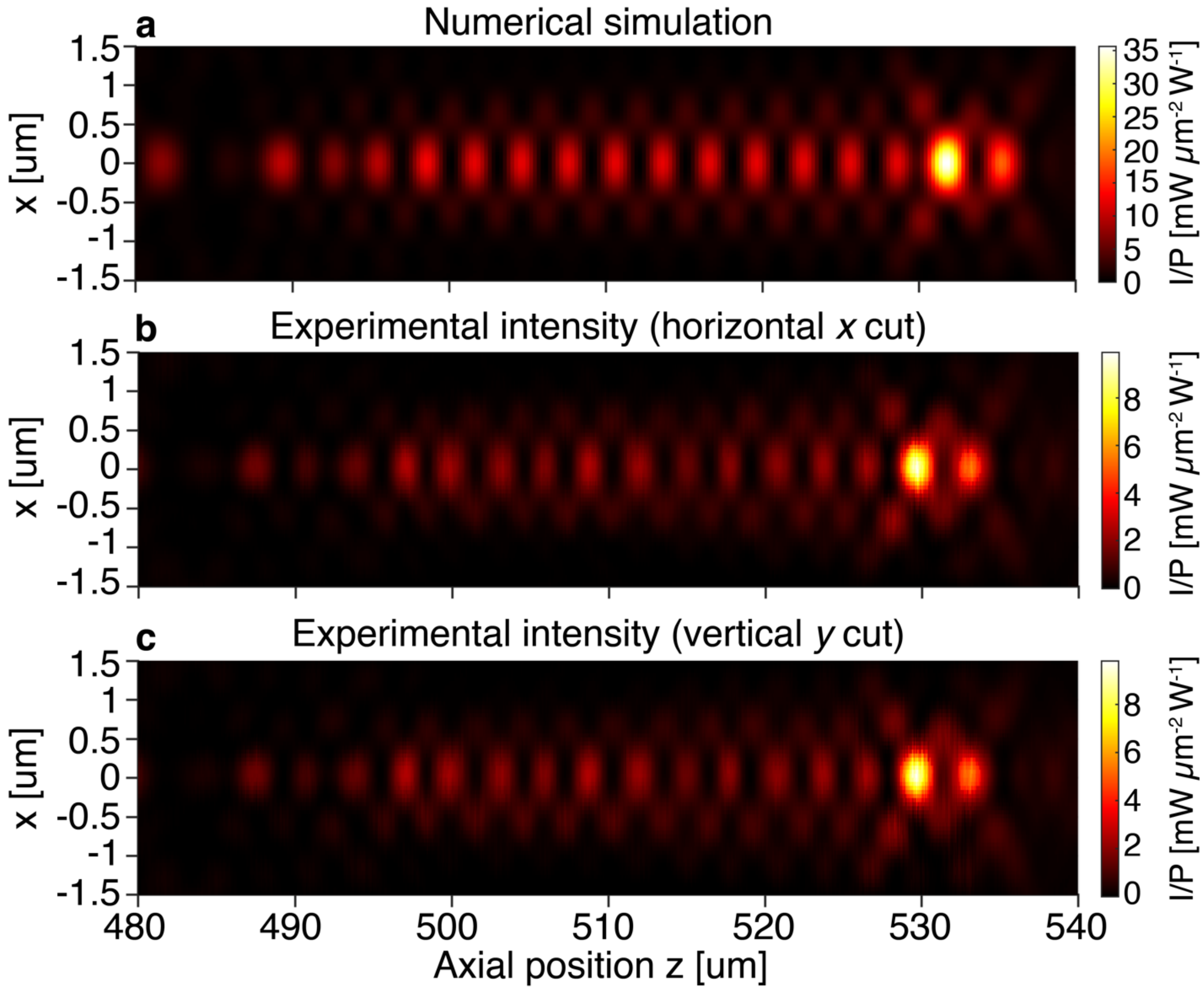

Supplementary Figure 14. Longitudinal intensity profile comparison between the numerical simulation and experiment. These images are identical to that of **Fig. 4a-c** in the main text with the colorbar adjusted to show the full dynamic range of intensities without saturation.

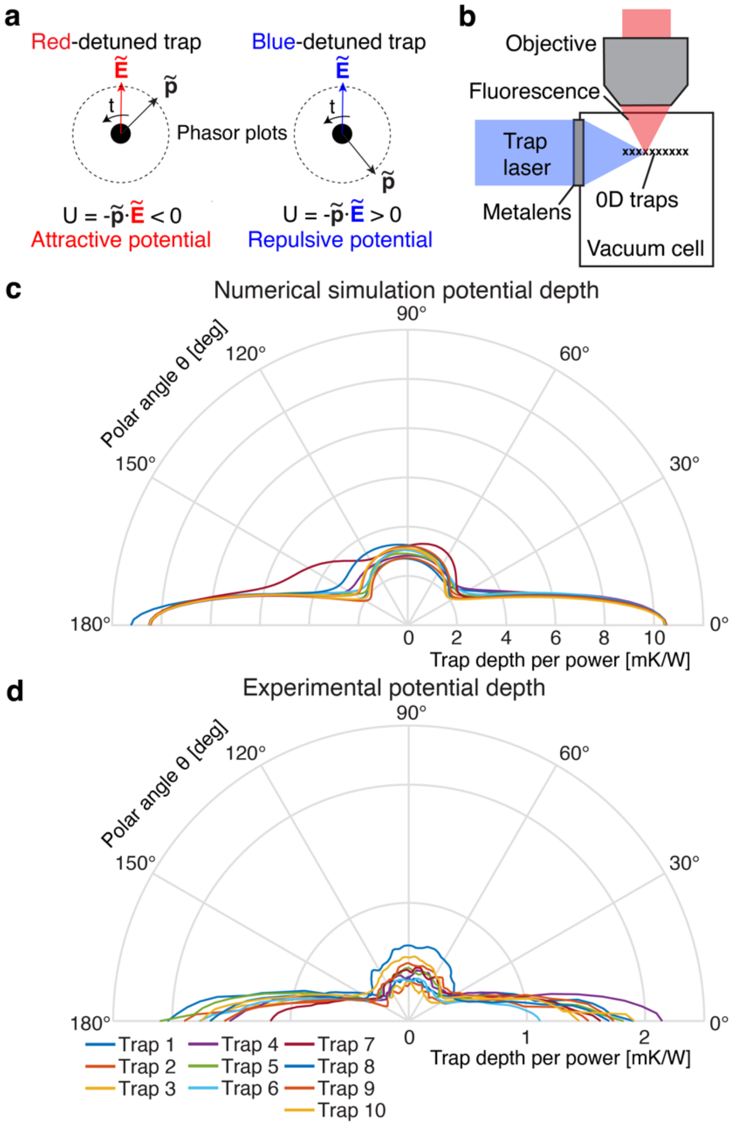

Supplementary Figure 15. Operation of 0D optical singularities as blue-detuned atom traps. (**a**) Time-domain phasor plots of the driving electric field **E** and driven electric dipole moment **p** for driving frequencies that are red and blue detuned from the dipole resonance. As time progresses, the phasors rotate counterclockwise. The phase angle magnitude between **E** and **p** for red-detuned frequencies is always less than π/2, leading to an attractive electric dipole potential. The phase angle magnitude is larger than π/2 for blue-detuned frequencies, leading to a repulsive potential. A dark spot surrounded by blue-detuned light serves as a blue trap for neutral atoms. (**b**) Possible vacuum cell configuration to trap and interrogate atoms that are trapped by the light field from a metasurface. (**c**) Numerically simulated potential depth for ^{87}Rb atoms placed at the 0D singular locations of the simulated light field in **Figure 4(a)**. The polar angle θ is the angle from the optical axis and the potential depth is expressed in temperature units of millikelvin per watt of incident light on the metasurface. (**d**) Potential depth for ^{87}Rb atoms placed at the 0D singular locations of the experimental light field in **Figure 4**(**b-c**).

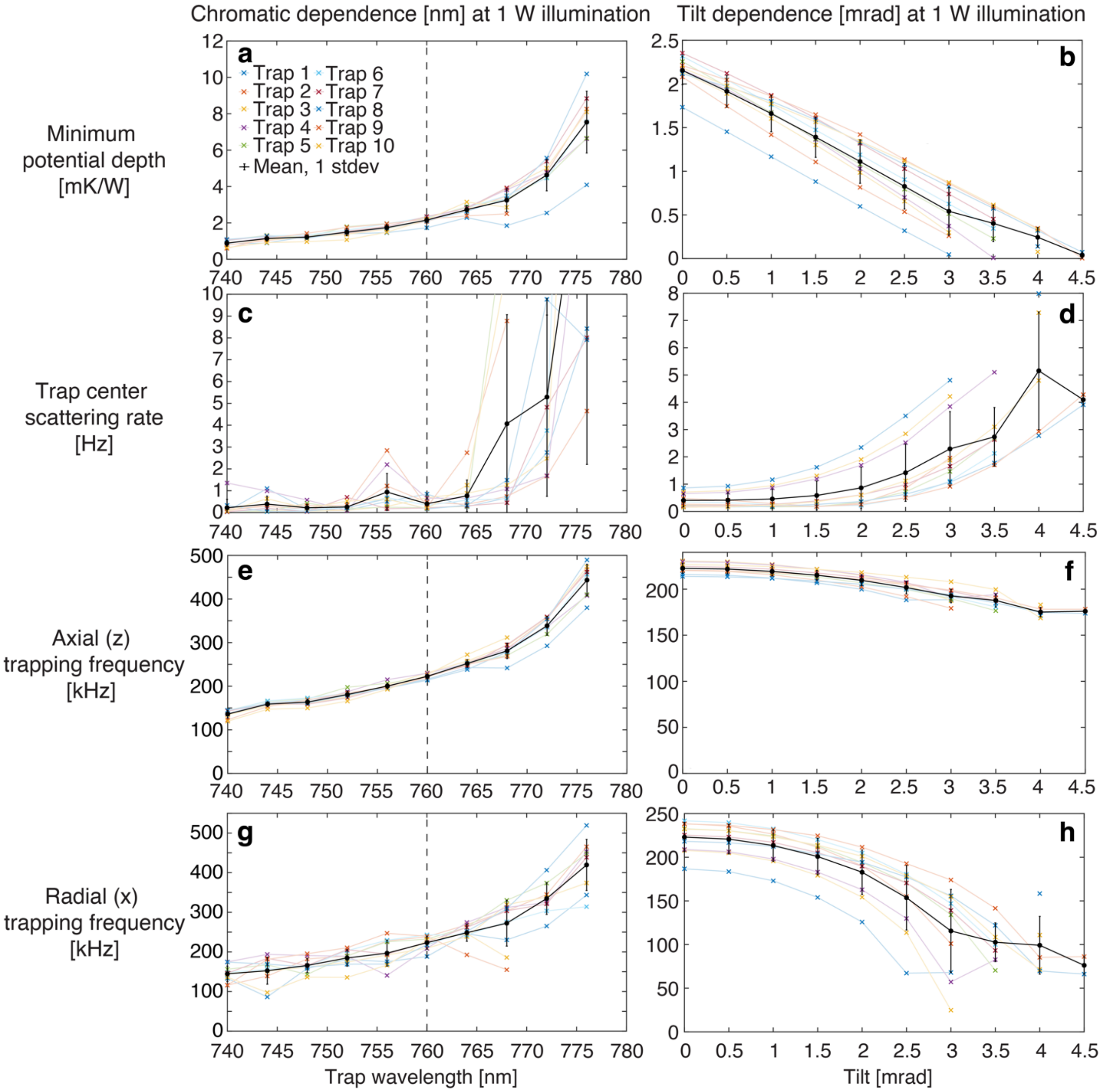

Supplementary Figure 16. Dependence of trapping parameters on wavelength and incident wavefront tilt for each of the ten 0D singularity traps generated by the metasurface used as blue traps for ^{87}Rb. All calculations assume 1W of incident power at the metasurface. Error bars indicate one standard deviation of variation over the ten traps. (**a**) Escape potential depth, (**c**) trap center scattering rate, (**e**) axial and (**g**) radial trapping frequencies as a function of incident wavelength. (**b**) Escape potential depth, (**d**) trap center scattering rate, (**f**) axial and (**h**) radial trapping frequencies as a function of incident tilt. The tilt is defined as the angle from the surface normal for the incident wavefront at the air/glass interface on the back face of the metasurface.

Supplementary References

1. Kuga, T. *et al.* Novel Optical Trap of Atoms with a Doughnut Beam. *Phys Rev Lett* **78**, 4713–4716 (1997).
2. Marathay, A. S. & McCalmont, J. F. Vector diffraction theory for electromagnetic waves. *Journal of the Optical Society of America A* **18**, 2585–2593 (2001).
3. Abadi, M. *et al.* TensorFlow: A System for Large-Scale Machine Learning. in *Proceedings of the 12th USENIX Symposium on Operating Systems Design and Implementation (OSDI '16)* 265–283 (2016).
4. Endres, M. *et al.* Atom-by-atom assembly of defect-free one-dimensional cold atom arrays. *Science* **354**, 1024–1027 (2016).
5. Hugonin, J. P. & Lalanne, P. Reticolo software for grating analysis. (2005).
6. Steck, D. A. *Rubidium 87 D Line Data*. http://steck.us/alkalidata (2021).
7. Zimmermann, B., Müller, T., Meineke, J., Esslinger, T. & Moritz, H. High-resolution imaging of ultracold fermions in microscopically tailored optical potentials. *New J Phys* **13**, 043007 (2011).
8. Lester, B. J., Luick, N., Kaufman, A. M., Reynolds, C. M. & Regal, C. A. Rapid Production of Uniformly Filled Arrays of Neutral Atoms. *Phys Rev Lett* **115**, 073003 (2015).
