# Supplementary material for: Point singularity array with metasurfaces
Source: Nat Commun. 2023 Jun 5;14:3237. doi: 10.1038/s41467-023-39072-6 (PMC10241946; doi:10.1038/s41467-023-39072-6)
Supplement: Supplementary file 3 — Description of Additional Supplementary Files [file 41467_2023_39072_MOESM3_ESM.pdf]

Description of Additional Supplementary Files

File Name: Supplementary Movie 1
Description: Variation of 0D singularity array field structure with changes in incident wavelength, linearly scaled (top) and logarithmically scaled (bottom). Trap positions are indicated with crosses and vanish when 3D confinement is lost.

File Name: Supplementary Movie 2
Description: Variation of 0D singularity array field structure with changes in incident beam tilt on the metasurface, linearly scaled (top) and logarithmically scaled (bottom). Trap positions are indicated with crosses and vanish when 3D confinement is lost.
